# Supplementary material for: FRET-based cyclic GMP biosensors measure low cGMP concentrations in cardiomyocytes and neurons
Source: Commun Biol. 2019 Oct 29;2:394. doi: 10.1038/s42003-019-0641-x (PMC6820734; doi:10.1038/s42003-019-0641-x)
Supplement: Supplementary file 1 — Supplementary Information [file 42003_2019_641_MOESM1_ESM.pdf]

Supplementary Figure 1

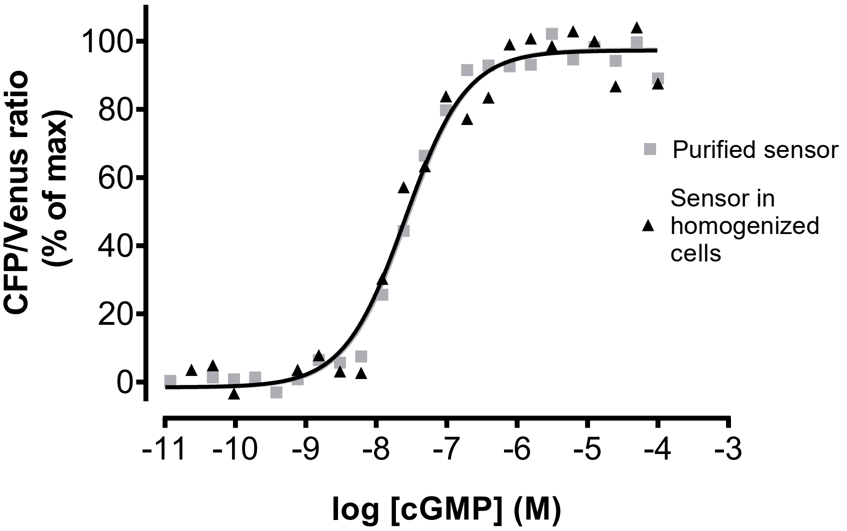

Purified Yellow *Pf*PKG biosensor retains the affinity for cGMP.

HEK293 cells expressing the His-TEV-*Pf*PKG biosensor were homogenized. 90 % of the homogenates was used to purify the biosensor as described in Methods and this, together with the remaining homogenate, were subjected to *in vitro* FRET assay with increasing concentrations of cGMP, as described in Methods. The EC_{50} was 27±2 nM and 30±2 nM for homogenized and purified sensor, respectively. This indicates that the potential influence of endogenous cGMP from HEK293 cells on the determined cGMP affinity of our biosensor is negligible. The graph shows data representative of 3 individual experiments.

Supplementary Figure 2

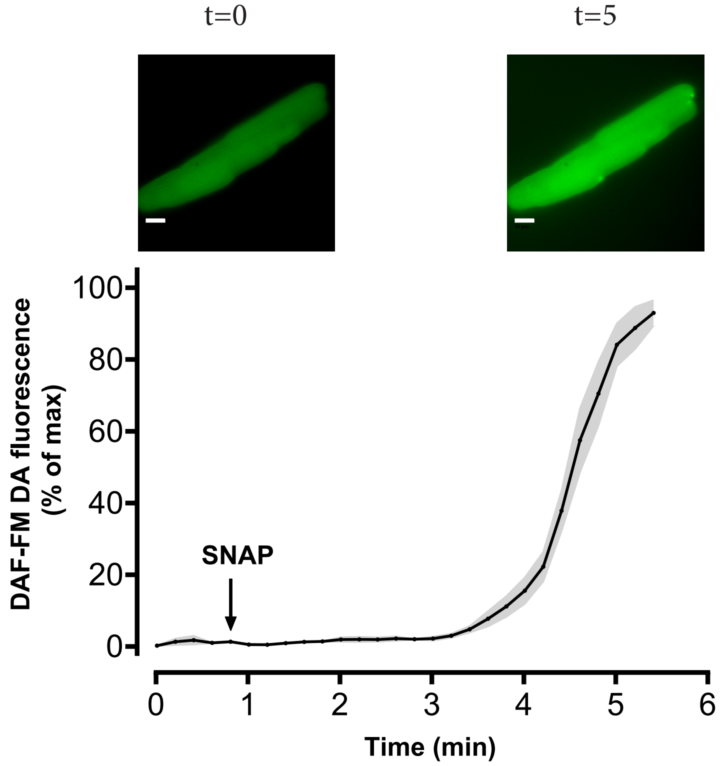

SNAP increases NO in cardiomyocytes.

Recording of fluorescence in cardiomyocytes loaded with DAF-FM DA, as described in Methods, stimulated with SNAP (100 µM) at the indicated time. The trace shows NO release within 5 min after SNAP addition. Data are mean±SEM of 6 cells. Images show fluorescence at the indicated time (min). Scale bar: 10 µm.

Supplementary Figure 3

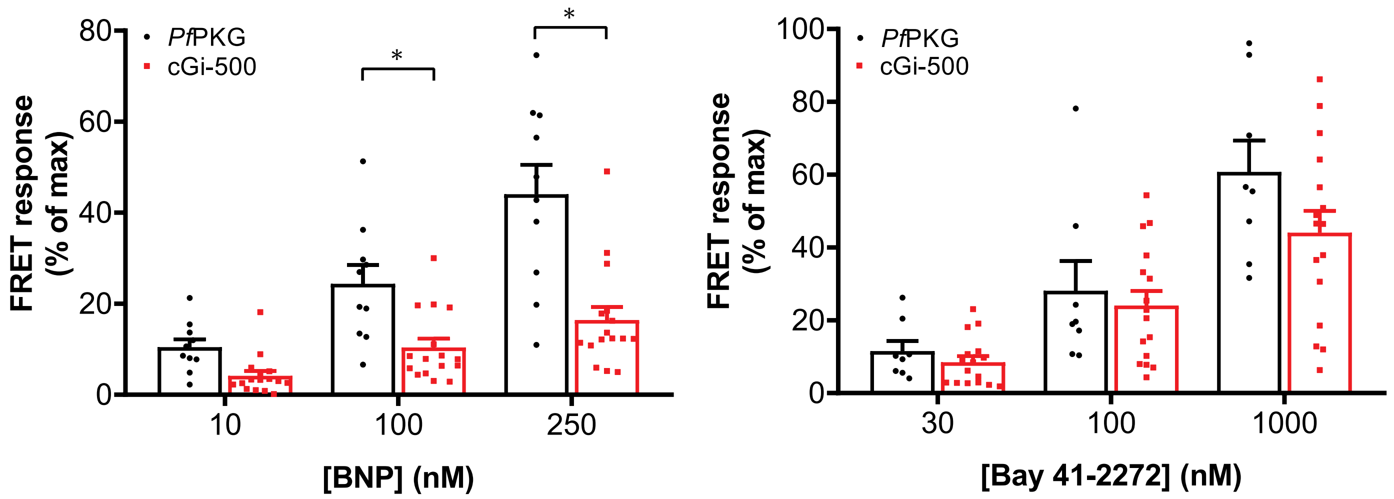

Yellow *Pf*PKG biosensor detects cGMP in SG neurons

Quantification of FRET reponses upon stimulation with increasing concentrations of BNP or Bay 41-2272 (from Figure 4a, b, d and e) comparing the reponses of the two biosensor as per cent of maximal stimulation (after SIN-1+IBMX). Data are mean±SEM. *$p<0.05$ cGi-500 vs. *Pf*PKG (Two-way ANOVA with Sidak's multiple comparisons test).
