# Supplementary material for: FRET-based cyclic GMP biosensors measure low cGMP concentrations in cardiomyocytes and neurons
Source: Commun Biol. 2019 Oct 29;2:394. doi: 10.1038/s42003-019-0641-x (PMC6820734; doi:10.1038/s42003-019-0641-x)
Supplement: Supplementary file 2 — Reporting Summary [file 42003_2019_641_MOESM2_ESM.pdf]

nature research

Corresponding author(s): Finn Olav Levy

Last updated by author(s): Sep 18, 2019

Reporting Summary

Nature Research wishes to improve the reproducibility of the work that we publish. This form provides structure for consistency and transparency in reporting. For further information on Nature Research policies, see Authors & Referees and the Editorial Policy Checklist.

Statistics

For all statistical analyses, confirm that the following items are present in the figure legend, table legend, main text, or Methods section.

n/a	Confirmed	
☐	☒	The exact sample size (*n*) for each experimental group/condition, given as a discrete number and unit of measurement
☐	☒	A statement on whether measurements were taken from distinct samples or whether the same sample was measured repeatedly
☐	☒	The statistical test(s) used AND whether they are one- or two-sided<br>*Only common tests should be described solely by name; describe more complex techniques in the Methods section.*
☒	☐	A description of all covariates tested
☐	☒	A description of any assumptions or corrections, such as tests of normality and adjustment for multiple comparisons
☐	☒	A full description of the statistical parameters including central tendency (e.g. means) or other basic estimates (e.g. regression coefficient) AND variation (e.g. standard deviation) or associated estimates of uncertainty (e.g. confidence intervals)
☐	☒	For null hypothesis testing, the test statistic (e.g. *F*, *t*, *r*) with confidence intervals, effect sizes, degrees of freedom and *P* value noted<br>*Give P values as exact values whenever suitable.*
☒	☐	For Bayesian analysis, information on the choice of priors and Markov chain Monte Carlo settings
☒	☐	For hierarchical and complex designs, identification of the appropriate level for tests and full reporting of outcomes
☒	☐	Estimates of effect sizes (e.g. Cohen's *d*, Pearson's *r*), indicating how they were calculated

Our web collection on statistics for biologists contains articles on many of the points above.

Software and code

Policy information about availability of computer code

Data collection	EnVision Software version1.12 (PerkinElmer, USA, NY, Melville);Olympus Fluoview 1000, Live Acquisition browser (FEI), OptoFluor CoolSNAP software (Cairn Research Ltd)
Data analysis	Microsoft Excel, GraphPad Prism 7 and 8 for Windows (GraphPad Software, San Diego, CA), Offline Analysis software (FEI)

For manuscripts utilizing custom algorithms or software that are central to the research but not yet described in published literature, software must be made available to editors/reviewers. We strongly encourage code deposition in a community repository (e.g. GitHub). See the Nature Research guidelines for submitting code & software for further information.

Data

Policy information about availability of data

All manuscripts must include a data availability statement. This statement should provide the following information, where applicable:

- Accession codes, unique identifiers, or web links for publicly available datasets
- A list of figures that have associated raw data
- A description of any restrictions on data availability

Nucleotide sequence for Yellow PfPKG and Red PfPKG have been deposited in GenBank. Accession numbers and sequences will become available to the public at the publication date.
The data that support the findings of this study are available from the corresponding author upon request.
The authors declare that the data supporting the findings of this study are available within the paper and its supplementary information files

Field-specific reporting

Please select the one below that is the best fit for your research. If you are not sure, read the appropriate sections before making your selection.

☒ Life sciences ☐ Behavioural & social sciences ☐ Ecological, evolutionary & environmental sciences

For a reference copy of the document with all sections, see nature.com/documents/nr-reporting-summary-flat.pdf

Life sciences study design

All studies must disclose on these points even when the disclosure is negative.

Sample size	A sample size of minimum 3 individual cells from at least two animals were considered minimum for experiments with a large effect and positive control. For experiments with large variation, a larger sample size was included to account for such variability.
Data exclusions	No data were excluded from the study.
Replication	Experiments were replicated with several biological replicates. This is indicated in the figure legend as number of cells from number of animals.
Randomization	Allocation was not random, as drug addition in the FRET experiments were sequential due to the nature of these experiments.
Blinding	Blinding was not possible due to sequential drug addition in the FRET experiments.

Reporting for specific materials, systems and methods

We require information from authors about some types of materials, experimental systems and methods used in many studies. Here, indicate whether each material, system or method listed is relevant to your study. If you are not sure if a list item applies to your research, read the appropriate section before selecting a response.

Materials & experimental systems

n/a	Involved in the study	
☒	☐	Antibodies
☐	☒	Eukaryotic cell lines
☒	☐	Palaeontology
☐	☒	Animals and other organisms
☒	☐	Human research participants
☒	☐	Clinical data

Methods

n/a	Involved in the study	
☒	☐	ChIP-seq
☒	☐	Flow cytometry
☒	☐	MRI-based neuroimaging

Eukaryotic cell lines

Policy information about cell lines

Cell line source(s)	HEK293 from ATCC
Authentication	the cell line used were not authenticated, but originated from ATCC
Mycoplasma contamination	HEK293 cell line tested negative for mycoplasma contamination
Commonly misidentified lines (See ICLAC register)	ICLAC does not have any information on HEK293 cells being misidentified.

Animals and other organisms

Policy information about studies involving animals; ARRIVE guidelines recommended for reporting animal research

Laboratory animals	300-400 gr Male Wistar rats were used
Wild animals	the study did not involve wild animals
Field-collected samples	the study did not involve samples collected from the field
Ethics oversight	Animal care was according to the Norwegian Animal Welfare Act (and approved by the Norwegian Animal Research Authority) and Use of Laboratory Animals (publication no. 85-23, revised 1996) and Animals (Scientific Procedures) Act 1986 (United

Kingdom) which conforms to the European Convention for the protection of Vertebrate animals used for Experimental and other Scientific Purposes (Council of Europe no. 123, Strasbourg 1985).

Note that full information on the approval of the study protocol must also be provided in the manuscript.
